# A Novel Position-Specific Encoding Algorithm (SeqPose) of Nucleotide Sequences and Its Application for Detecting Enhancers

**DOI:** 10.3390/ijms22063079

**Published:** 2021-03-17

**Authors:** Xuechen Mu, Yueying Wang, Meiyu Duan, Shuai Liu, Fei Li, Xiuli Wang, Kai Zhang, Lan Huang, Fengfeng Zhou

**Affiliations:** 1 Health Informatics Lab, College of Computer Science and Technology, Key Laboratory of Symbolic Computation and Knowledge Engineering of the Ministry of Education, Jilin University, Changchun 130012, China; m250921296@gmail.com (X.M.); Wyy18@mails.jlu.edu.cn (Y.W.); dmy235813@163.com (M.D.); shuailiu2021@gmail.com (S.L.); comeherewinter@gmail.com (F.L.); huanglan@jlu.edu.cn (L.H.); 2 School of Mathematics, Jilin University, Changchun 130012, China; xiuli19@jlu.edu.cn (X.W.); zhangkaimath@jlu.edu.cn (K.Z.); 3 Department of Epidemiology and Biostatistics, School of Public Health, Jilin University, Changchun 130021, China

**Keywords:** two-layer classification model, position-coding, feature selection, text vectorization, attention mechanism

## Abstract

Enhancers are short genomic regions exerting tissue-specific regulatory roles, usually for remote coding regions. Enhancers are observed in both prokaryotic and eukaryotic genomes, and their detections facilitate a better understanding of the transcriptional regulation mechanism. The accurate detection and transcriptional regulation strength evaluation of the enhancers remain a major bioinformatics challenge. Most of the current studies utilized the statistical features of short fixed-length nucleotide sequences. This study introduces the location information of each k-mer (SeqPose) into the encoding strategy of a DNA sequence and employs the attention mechanism in the two-layer bi-directional long-short term memory (BD-LSTM) model (spEnhancer) for the enhancer detection problem. The first layer of the delivered classifier discriminates between enhancers and non-enhancers, and the second layer evaluates the transcriptional regulation strength of the detected enhancer. The SeqPose-encoded features are selected by the Chi-squared test, and 45 positions are removed from further analysis. The existing studies may focus on selecting the statistical DNA sequence descriptors with large contributions to the prediction models. This study does not utilize these statistical DNA sequence descriptors. Then the word vector of the SeqPose-encoded features is obtained by using the word embedding layer. This study hypothesizes that different word vector features may contribute differently to the enhancer detection model, and assigns different weights to these word vectors through the attention mechanism in the BD-LSTM model. The previous study generously provided the training and independent test datasets, and the proposed spEnhancer is compared with the three existing state-of-the-art studies using the same experimental procedure. The leave-one-out validation data on the training dataset shows that the proposed spEnhancer achieves similar detection performances as the three existing studies. While spEnhancer achieves the best overall performance metric MCC for both of the two binary classification problems on the independent test dataset. The experimental data shows that the strategy of removing redundant positions (SeqPose) may help improve the DNA sequence-based prediction models. spEnhancer may serve well as a complementary model to the existing studies, especially for the novel query enhancers that are not included in the training dataset.

## 1. Introduction

The innovative technologies and comprehensive biological investigations show that the non-coding genomic regions are not functionally inactive as previously hypothesized and play essential roles in transcriptional regulations [1]. An enhancer is a small genomic region that binds to transcription factors and exerts its regulatory roles to the target genes [2,3]. Enhancers are different from promoters that are always upstream to the target genes and may even reside in the introns [4]. The burst frequency of a target gene may be significantly increased by enhancers [5]. Therefore, the functional investigation of enhancers will improve our understanding of the transcription regulation mechanism [6].

Enhancers may be detected through in vivo animal experiments. Heintzman and Ren identified novel enhancers through the binding affinities to the transcription factors like *P300* [7]. Boyle et al. detected enhancers by investigating the *DNaseI* hypersensitivity [8]. However, the wet-lab experiments are time-consuming and labor-intensive, and many enhancers cannot be detected in this way due to their condition-specific activities [9].

Various machine learning methods are proposed for the enhancer detection and evaluation problem. Firipi et al. proposed the artificial neural network-based algorithm CSI-ANN to efficiently extract the enhancers’ sequence features and accurately detect novel ones [10]. The tool EnhancerFinder combined multiple learning kernels based on the evolutionary conservation patterns, sequence motifs, and cell type-specific functional information for the detection, and genomic distribution characterization of enhancers [3]. A random forest (RF) model was trained using the chromatin status to construct the enhancer maps in multiple cell types [11]. Deep learning is another powerful tool to detect enhancers, and the tool EnhancedDBN achieved the enhancer detection task using a deep belief network (DBN) [12].

The strength type is another important feature of enhancers. Liu et al. utilized the pseudo-k-set nucleotide composition (PseKNC) algorithm [13] as the sequence features and proposed a two-layer classifier iEnhancer-2L for both the enhancer detection and the enhancer strength type determination [14]. Jia et al. utilized a two-step wrapper feature selection algorithm to find the best features from the useful information of bot bi-profile Bayes and PseKNC, and their model EnhancerPred by 0.01 and 0.12 in the metric Matthews Correlation Coefficient (MCC) for the two layers of iEnhancer-2L [15]. Nguyen et al. combined the one-hot-encoding of and the statistical k-mer descriptors neighboring to each nucleotide, and trained convolutional neural network (CNN) models for the enhancer detection problem (iEnhancer-ECNN) [16]. Their ensembled models demonstrated much improved prediction performance on the independent test dataset. Both the ensembled prediction models and the CNN classifiers are notorious for the high computational requirements. This might be the reason that the leave-one-out validation was not carried out.

This study proposes a novel position-specific encoding algorithm (SeqPose) of nucleotide sequences and selects a subset of the SeqPose features to build the two-layer enhancer classification model. The first layer separates the enhancers from the non-enhancers, and the second layer determines whether an enhancer is strong or weak. The experimental data shows that the position-specific patterns contribute useful information to the accurate enhancer detection and strength evaluation problem.

There are two major contributions of this study. Firstly, the DNA sequence encoding strategy in this study utilizes the location information of each k-mer. In this paper, a novel sequence preprocessing strategy (SeqPose) is proposed. We map the original DNA sequences into numerical sequences by using the encoding rules (SeqPoseDict), and then select the redundant positions with low correlation with sample label by using the Chi-squared test, and remove them from the sequences altogether. In this study, we look at the different positions of the encoded sequence as features, trying to find unimportant positions and delete them from the sample, which was ignored by most of the existing studies. The correlations between different positions in a DNA sequence also shows phenotype associations. This is different from the previous position-specific encoding strategy of DNA sequences, which calculates the one-hot encoding of and the other statistical descriptors neighboring to the nucleotide A/C/G/T in each position and formulates these engineered features in the same orders of their corresponding nucleotides [16].

Secondly, the attention mechanism is widely used in the deep learning-based text classification studies, and this study hypothesizes that the attention mechanism might be able to highlight the nucleotide ‘words’ with large contributions to the enhancer prediction models. Our experimental data in the following sections demonstrates that the proposed SeqPose features achieves similar leave-one-out validation and independent test performances compared with the existing studies. The feature selection strategy and the attention mechanism ensure that the training and prediction of the proposed prediction models may be completed within reasonable time and have the potential to be deployed to the situations with limited computing power.

## 2. Results and Discussion

This section evaluates the parameters of the proposed model spEnhancer, and then compares spEnhancer with the existing studies on the same datasets. In the first three subsections, we divide 10% of the 2968 training DNA sequences into test sets, then 10% of the remaining data sets into verification sets, and the rest were all used as training sets for training models. All experiments in this study the random number seed 75. The results of the first layer structure of the model on the test set are used as the criteria for parameter selection.

### 2.1. Evaluating the Length of K-mers

It is anticipated that different length of k-mers makes different SeqPose features and may have large impacts on the final prediction models. This section investigates the binary classification between enhancers and non-enhancers. The three parameters are initially set as pBatchSize = 100, pLSTMSize = 128, and pDropoutRatio = 0.2.

The enhancer detection model of 7-mers achieves the worst prediction accuracy (Acc), as shown in Table 1. The data suggests that the model of 6-mers performed reasonably well on the metric specificity (Sp), but its sensitivity (Sn) is only 0.6338, which is at least 0.0775 worse than the models using the other k-mers. Therefore, 6-mers are excluded from further evaluation. The data suggests that the model using 2-mers performed the best Acc = 0.8047 in Table 1. The following sections focus on the models using 2-mers.

### 2.2. Selecting the Best SeqPose Features

**Figure 1:**
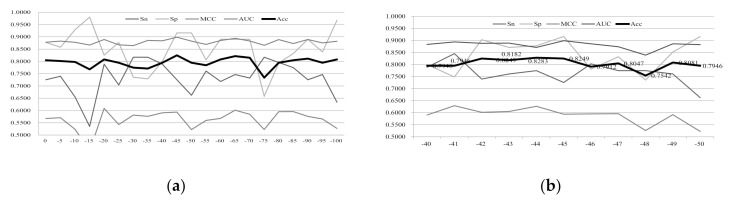
Performance evaluation of the models using the 2-mer SeqPose features after filtering out some redundant features. The vertical axis gives the values of the five performance metrics, including Sn, Sp, MCC, AUC, and Acc. The horizontal axis is the number of features removed from the 2-mer SeqPose features, i.e., “−5” means that the five features with the largest *p*-values are removed from the list of all 2-mer SeqPose features. The feature removal is carried out in two steps, i.e., (**a**) five features are removed in each iteration; and then (**b**) one feature is removed in each iteration from the list of features “−45”.

We hypothesize that some of the SeqPose features had no contributions to the classification problem in this study. We use the Chi2 measurement to evaluate the features and calculate the prediction performances of the binary classification problem between enhancers and non-enhancers on the test dataset after removing some features, as shown in Figure 1.

Firstly, we carry out a procedure of coarse-grain feature selection, through removing five features with the largest *p*-value measurement in each iteration, as shown in Figure 1a. There are 398 2-mer SeqPose features constructed from each 200-bp nucleotide sequences. The best classification accuracy is Acc = 0.8249 after removing 45 features (“−45”). At the same time, the classification model based on the feature list also obtains the best parameter-independent metric AUC = 0.8986.

Secondly, we carry out a fine-grain feature selection procedure by removing one feature in each iteration, as shown in Figure 1b. The experimental data suggests that the classification model with 45 removed features is not improved with more features being removed. The experimental data of Table 1 and Figure 1 show that 45 of the SeqPose features do not contribute to the enhancer prediction models. The best prediction model in Table 1 was substantially improved for Acc (from 0.8047 to 0.8249), MCC (from 0.5673 to 0.5936), and AUC (from 0.8781 to 0.8986). Therefore, the removal of the redundant positions in the SeqPose encoding features may improve the DNA sequence-based prediction models like the enhancer predictions. The following section will use the 2-mer SeqPose features after the removal of those 45 position-specific features.

### 2.3. Symbolic Interpretation of SeqPoseDict

This section demonstrates through experimental comparison that SeqPoseDict is only used to convert DNA sequences into computer-recognizable numerical representations, and its selection will not have a decisive impact on the final experimental results (see Table 2). We sequentially: (1) use all data sets to construct SeqPoseDict, see Res1; (2) use 95% of all data sets to construct SeqPoseDict, see Res2; (3) use 90% of all data sets to construct SeqPoseDict, see Res3; (4) use 85% of all data sets to construct SeqPoseDict, see Res4; (5) use 80% of all data sets to construct SeqPoseDict, see Res5.

The data shows that under the framework proposed in this paper, the size of SeqPoseDict does not have a decisive influence on the prediction effect of the model. It can be seen that when the data set used to construct SeqPoseDict fluctuates within 20%, the range of the Acc indicator does not exceed 0.03, which is within the acceptable range.

### 2.4. Optimizing the Best Choices of the Three Parameters

The three parameters pLSTMSize, pDropoutRatio, and pBatchSize are optimized through eight-fold cross-validation, as shown in Table 3. The overall prediction accuracy Acc is used as a parameter selection goal for the binary classification problem between enhancers and non-enhancers.

The set of pLSTMSize is $\left\{64,128,192\right\}$; the set of pDropoutRatio is $\left\{0.1,0.2,0.3,0.4,0.5,0.6,0.7\right\}$; the set of pBatchSize is $\left\{16,32,64,128,256,512\right\}$. Every time we take out a set of hyperparameters from these three sets as training parameters, until we get all possible combinations, compare their evaluation indicators on the same test set (See Table 3). The data shows that when the hyperparameter combination is pLSTMSize = 64, pBatchSize = 64, and pDropoutRatio = 0.5, the model training effect is the best (Acc = 0.7884, AUC = 0.8506). Therefore, the following sections are conducted on the basis of this set of hyperparameters.

### 2.5. Comparing spEnhancer with the Existing Models

This section compares the experimental results of the proposed spEnhancer models with the existing models, as shown in Table 4 and Table 5. The parameters of spEnhancer models are set by the optimization procedure in the above sections. The SeqPose encoding features use 2-mers. The 45 redundant positions are removed according to the above sections. The parameters pLSTMSize, pBatchSize, and pDropoutRatio are set as 64, 64, and 0.5, respectively. The random seed is set to 75. The SeqPoseDict is calculated based on the training dataset. The threshold of the prediction probability uses the default value 0.5.

The performance metrics Sn and Sp measure a binary classification model from different aspects, and one metric may be increased at the cost of the other one [17,18]. The overall prediction performance metrics Acc and MCC may be used for a fair comparison of two classification models [14]. Leave-one-out validation tends to give optimistic results for a classification model and the model generalization on future samples is usually evaluated by the prediction performances on independent datasets that are not involved in the model training process. The same independent dataset is publicly available and used in this study [14].

Table 4 shows that the proposed method spEnhancer achieves similar prediction performances for both of the two binary classification problem using the leave-one-out validation. The spEnhancer model achieves Acc = 0.7793 for the classification problem between enhancers and non-enhancers, which is 0.001 smaller than the best accuracy of the iEnhancer-EL model. Due to the difference in experimental equipment and the influence of the randomness of the parameters, we consider the difference in accuracy to be negligible and acceptable. The spEnhancer model achieved the second-best accuracy Acc = 0.6413, which is slightly smaller than the best model iEnhancer-EL’s Acc = 0.6503.

Table 5 gives the comparative results between the proposed spEnhancer and the four existing state-of-the-art models on the independent test dataset. The prediction performances of the four existing state-of-the-art studies are also retrieved from the previous study. The prediction accuracy on the independent dataset is supposed to represent how well a prediction model may achieve on the future query nucleotide sequences.

The proposed spEnhancer models and another algorithm iEnhancer-ECNN achieve the best two prediction accuracies and MCC on both enhancer detection problems, as shown in Table 5. The spEnhancer model achieves the best prediction Acc = 0.7725 and MCC = 0.5793 for the first layer of the enhancer detection problem, suggesting that spEnhancer may accurately separate enhancers from non-enhancers even on novel query sequences. For the second layer of the enhancer detection problem, spEnhancer achieves Acc = 0.6200, which is slightly worse than the Acc = 0.6780 of the iEnhancer-ECNN model [16]. However, spEnhancer outperforms iEnhancer-ECNN in MCC by an improvement of 0.0023.

The previously best model iEnhancer-ECNN achieves a better strong/weak enhancer prediction accuracy than the proposed spEnhancer with a sacrifice in the training and prediction time. The iEnhancer-ECNN integrates the prediction results of five convolutional neural networks, each of which is trained by 20 epochs [16]. While this study completes the training of the proposed spEnhancer within six epochs. This might be the reason that the iEnhancer-ECNN model does not provide the leave-one-out validation results.

Therefore, the overall prediction performances of spEnhancer are better than or comparable to the four state-of-the-art models. This suggests that spEnhancer may serve well as a complementary model to the existing enhancer detection studies.

### 2.6. Evaluating the Three-Class Classification Model

The BD-LSTM algorithm may also handle the three-class classification problem. The above sections formulate the enhancer detection problem as a two-layer setting for a consistent comparison with the network structure of Liu et al. [17]. This section collects the three classes of samples and directly trains a three-class BD-LSTM prediction model with the same parameter values for the enhancer detection problem. The model training is on the training dataset and the performance is calculated using the independent test dataset. The three-class BD-LSTM model achieves 0.6402 in the overall prediction accuracy, while the binary classification BD-LSTM model achieves Acc = 0.7725 for separating enhancers from non-enhancers, as shown in Table 5. However, the three-class BD-LSTM model outperforms the binary classification model between strong and weak enhancers.

Therefore, the setting of the two-layer model delivers similar prediction performances as the three-class classification model. In order to carry out a direct comparison with the existing studies, the two-layer model is recommended.

### 2.7. Evaluating Different Word Vector Dimensions

The dimension size of the word vectors may affect the prediction performances, and this study evaluates different dimensions of the word vectors, as shown in Table 6. Eight values are evaluated for the dimension of the word vector, i.e., 12, 24, 48, 96, 192, 394, 768, and 1536. The values 48, 768, and 1536 achieve the best three Acc and MCC for the binary classification problem between enhancers and non-enhancers. The values 48, 96, 394, and 768 achieve the best four Acc for separating the strong enhancers from the weak ones, but the best MCC = 0.3703 is achieved by the dimension of word vector 768. At the same time, it can be seen that although the Acc with vector dimension equal to 1546 reached the optimal when classifying the enhancers and non-enhancers, the result was only 0.0039 higher than that of the Acc with dimension of 768. However, when classifying strong enhancers and weak enhancers, the Acc with a vector dimension of 768 was higher than the Acc with a vector of 1536, which was 0.04, which was a significant improvement. Therefore, this study sets 768 as the dimension of the word vector.

## 3. Materials and Methods

This section introduces a two-layer classifier for the detection and strength determination of enhancers. Firstly, the position-specific encoding algorithm (SeqPose) of nucleotide sequences is described. Then the Chi-squared test (Chi2) is used to select a subset of the SeqPose features. Lastly, the selected SeqPose features are loaded to the embedding layer and mapped to a three-dimensional tensor. The two-layer classifier spEnhancer is optimized for the two binary classification problems.

### 3.1. Datasets and Performance Metrics

This study retrieves the publicly available training and independent test datasets released by the previous study [14]. Three existing state-of-the-art models are evaluated using the same datasets. Therefore, the proposed model spEnhancer is also evaluated on these two datasets. A fair comparison is carried out for the proposed model spEnhancer and the three existing models using the leave-one-out validation on the training dataset and the validation on the independent test dataset. The following describes how the training dataset is generated. The independent test dataset is generated using the same procedure and has no overlapped samples with the training dataset.

This article cites the standard data set S constructed by Prof. Bin Liu [14] based on nine kinds of cell chromosome status information (2968 sequences in total)

(1) $$S=S_{strong}^{+}+S_{weak}^{+}+S^{-}$$

where, $\;S_{strong}^{+}$ contains only strong enhancer sequences, with a total of 742; $S_{weak}^{+}$ contains only weak enhancer sequences, with a total of 742; $S^{-}$ contains only non-enhancer sequences, with a total of 1484.

All data consists of a positive sample data set and a negative sample data set. Among them, the enhancer type in the first layer structure is a positive sample, and the non-enhancer type is a negative sample; the strong enhancer type in the second layer structure is a positive sample, and the weak enhancer type is a negative sample.

This study uses the five widely-used classification performance metrics [13], i.e., accuracy (Acc), Matthews correlation coefficient (MCC), sensitivity (Sn), specificity (Sp), and the area under the ROC curve (AUC). Let the samples with the class labels 1 and 0 be positive and negative ones, respectively. The metrics Sn and Sp describe the ratios of correctly predicted positive and negative samples, respectively. Acc is the ratio of the correctly predicted samples. MCC describes the correlation between the real and predicted class labels. AUC is a threshold-independent metric for a classification model, and a good model tends to have a large AUC value [19]. These five metrics are defined in the following formula.

(2) $$Sn=1-S_{-}^{+}/S^{+}$$

(3) $$Sp=1-S_{+}^{-}/S^{-}$$

(4) $$Acc=1-\left(S_{-}^{+}+S_{+}^{-}\right)/\left(S^{+}S^{-}\right)$$

(5) $$MCC=\frac{1-\left(S_{-}^{+}+S_{+}^{-}\right)/\left(S^{+}+S^{-}\right)}{\sqrt{\left[1+\left(S_{+}^{-}-S_{-}^{+}\right)/S^{+}\right]\left[1+\left(S_{-}^{+}-S_{+}^{-}\right)/S^{-}\right]}}$$

The notation $S^{+}$ is the number of positive samples, while $S_{-}^{+}$ is the number of incorrectly predicted positive samples. $S^{-}$ is the number of negative samples, and $S_{+}^{-}$ is the number of incorrectly negative samples.

### 3.2. K-mer Indexing and SeqPose Feature Extraction

**Figure 2:**
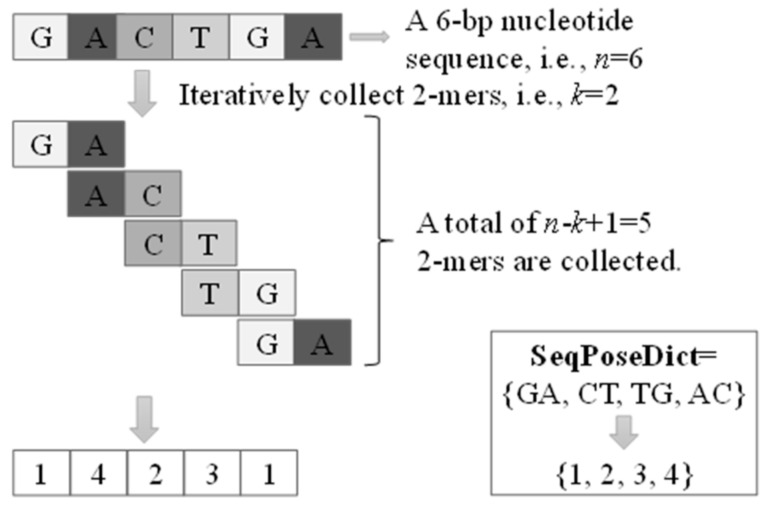
Extracted SeqPose features of a nucleotide sequence. A, T, G, C represent the bases in the nucleotide sequence.

This study hypothesizes that the position-specific k-mer patterns may deliver important information to discriminate enhancers from the other nucleotide sequences. A nucleotide sequence is a vector of letters and may be formulated as the one-hot integer vectors. Another feature extraction strategy is to summarize the statistical metrics of k-mers in a nucleotide sequence. All the k-mer instances are usually collected through a sliding window with step size 1, as shown in Figure 2.

The collected list of unique k-mers from all the training sequences is shuffled into a set, which is used as an enhancer dictionary (denoted as SeqPoseDict). Each k-mer has a unique ID (starting from 1) in this dictionary, SeqPoseDict. A query nucleotide sequence is encoded by the same dictionary generated from the training sequences. Note that SeqPoseDict ID is not in the actual meaning. It is used to map the DNA sequence for numerical vectors, we will in the next chapter by the experimental results show that different SeqPoseDict model does not cause large fluctuations, it also illustrates the model of our proposed architecture as a whole is robust. Here we only give one of the methods of constructing SeqPoseDict: according to the order of appearance of k-mer in the training set, assign values from 1 until all k-mers in the training set are traversed. For k-mers that are not in the SeqPoseDict in the unknown data set, we specify that the k-mer ID is 0.

### 3.3. Selecting the Subset of Best Features

A feature selection step is carried out to remove the positions extracted in the above section if these have low phenotype associations. Chi2 assumes the null hypothesis that under the Chi-square distribution, a given feature is independent of the class label. A statistical *p*-value is calculated to describe the null hypothesis. A small *p*-value rejects the null hypothesis and supports that the given position is correlated with the class label. Next, use Chi2 to determine the position label with low correlation with the label on the training set (T) as the redundant feature and remove it from all samples, including the test set.

**Figure 3:**
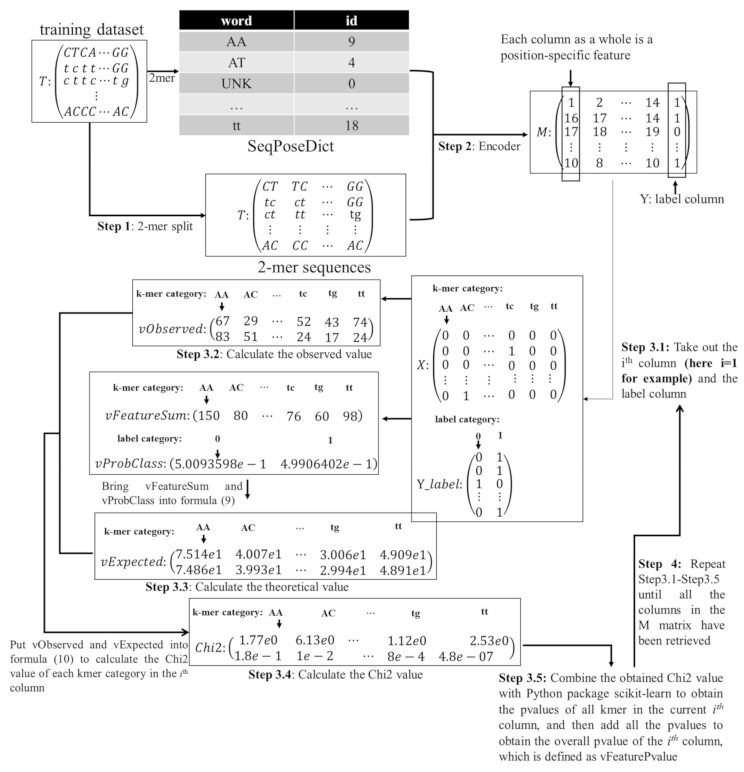
Flow chart of Chi2-based SeqPose feature selection.

Each sample is a fixed-length (n) nucleotide sequence and is sliced as multiple k-mers through the sliding window with step size 1. Each k-mer is regarded as a ‘word’. Therefore, each sample can be viewed as a ‘sentence’ composed of multiple ‘words’ (See step 1 in Figure 3).

The k-mer ‘word’ in the extracted feature of a query nucleotide sequence is replaced by its ID in the dictionary SeqPoseDict (See step 2 in Figure 3).

The samples in the training dataset and the labels of these corresponding samples are formed into a matrix M. That is, the first C-1 column of M indicates position-specific feature, the last column is the label column, and each row of M is a sample. The correlation between each feature and the class label is calculated by Chi2. The detailed calculations are described in detail in the following sub-steps.

Take out the ith column and the label column Y in M. We can treat the different IDs in the ith column as different k-mer categories and extend the column vectors into matrix X by one-hot coding, where each row represents the one-hot encoding of IDs, and each column represents the k-mer category. Then do the same for the label variable Y with one-hot coding to make a two-dimensional matrix Y_label, where the rows represent the one-hot code of the sample’s class label, and the column represents the sample’s class label (see Step 3.1 in Figure 3).

Calculate the observed value by formula (6) and record it as vObserved (see Step 3.2 in Figure 3).

(6) $$vObserved=Y^{T}X$$

Summing each column of the data matrix X makes the variable vFeatureSum. Then the formulas (7) and (8) are used to calculate the proportion of positive (vProbP) and negative (vProbN) samples in the column vector Y_label, respectively. The variables vProbP and vProbN are combined as vProbClass. The theoretical value is calculated according to the formula (9), denoted as vExpected. Please be noted that the calculation uses floating-point values with eight digits after the decimal point. Figure 3 shows only two digits after the decimal point for an easy view (see Step 3.3 in Figure 3).

(7) $$vProbP=\frac{N_{1}}{N}$$

(8) $$vProbN=\frac{N_{0}}{N}$$

(9) vExpected=vProbClassT×vFeatureSum

where *N*_1_, *N*_0_, and *N* represent the numbers of the samples in category 1, the samples in category 0 and all the samples, respectively.

Formula (10) calculates the Chi2 value of each k-mer category in a column of the data matrix M. Please be noted that the calculated Chi2 values reach 16 digits after the decimal point. Figure 3 rounds these values for a better view (see Step 3.4 in Figure 3).

(10) $$\mathrm{Chi}2=\frac{{\left(vObserved-vExpected\right)}^{2}}{vExpected}$$

The corresponding *p*-value is calculated using the tool Python package scikit-learn version 0.20.3. This paper defines the *p*-value of each column in the matrix M as the sum of *p*-values of each k-mer in this column, denoted as vFeaturePvalue (See step 3.5 in Figure 3).

Repeat the steps 3.1–3.5 until all the feature columns are evaluated (See step 4 in Figure 3).

The features are sorted by vFeaturePvalue in the descendent order, and the top K features with the largest vFeaturePvalue are removed for their small correlations with the class labels.

### 3.4. Vectorization

The features of a sample nucleotide sequence from the above section form a list of consecutive k-mer ‘words’, and this list is regarded as a ‘sentence’. The traditional one-hot coding strategy makes the feature matrix very sparse, so the step of text vectorization is carried out through the word embedding technique. A numeric tensor is generated by the text vectorization of a sample (a nucleotide sequence).

The dimension of the original one-hot coding feature vector of each k-mer is 1×P, and we project each k-mer into a Q-dimensional word vector by embedding layer. P is also the size of the dictionary *SeqPoseDict*. This paper uses the word embedding layer to map each k-mer to a word vector of Q dimensions. The recently proposed BERT model performs very well on the text-based classification problems. After evaluating eight values {12, 24, 48, 96, 192, 394, 768, 1536} for the dimension of word vectors, this study uses the same parameter settings of the token embedding layer from the BERT_BASE_ model [20], which assigns Q = 768. The detailed experimental data may be found in the following sections.

**Figure 4:**
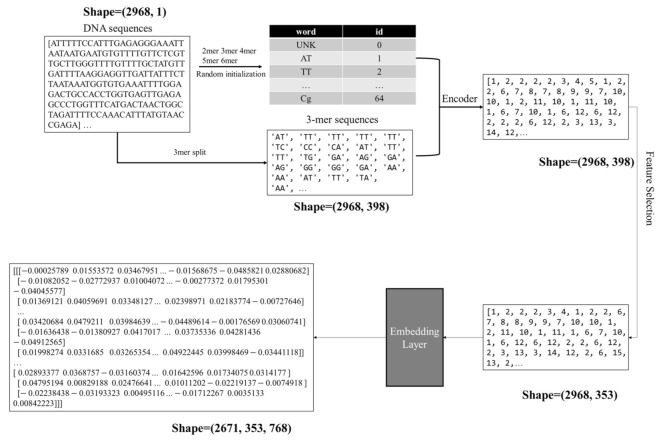
Illustration of the nucleotide sequence vectorization process.

The word embedding layer is randomly initialized and updated during the training process of the deep learning classifier. After each word passes through the word embedding layer, it will change from an ID value to a 768-dimensional row vector, as shown in Figure 4. This step generates the SeqPose features from the samples.

### 3.5. Structural Description of the Classifier SpEnhancer

**Figure 5:**
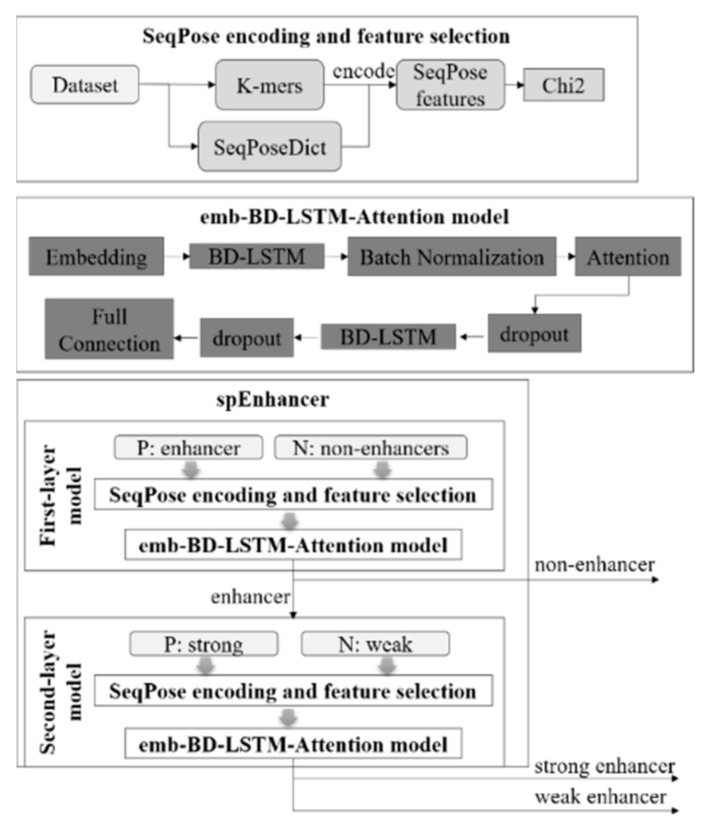
Workflow of the proposed two-layer model spEnhancer. A schematic diagram depicting the steps of feature extraction, feature selection, and prediction.

The two-layer enhancer prediction model spEnhancer is illustrated in Figure 5. All the nucleotide sequences are converted to the SeqPose features. Both of the two layers of the proposed classifier spEnhancer use the bi-directional long-short term memory (BD-LSTM) model combined with attention mechanism as the classifier because that the reverse complementary nature of a DNA molecule. The output of each neuron in the BD-LSTM is calculated from the input of the current neuron and its two neighboring neurons, and then passed as the input of batch normalization. The reason for introducing batch normalization is to prevent the problem of gradient disappearance. In addition, when extracting features, we hope that the model will pay more attention to features with high importance. Therefore, we introduce the attention mechanism in the model to assign weight coefficients to features to indicate the importance of the features, and then linearly combine the weight coefficients with the original input features and output to the next layer. The dropout layer is then utilized to mask a certain proportion of neurons from calculations and may effectively prevent over-fitting. This BD-LSTM layer delivers its output to the dropout layer, and then the full connection layer generates the final prediction results using one neuron with the value 1 or 0. The two layers of the proposed model spEnhancer have the same neural network architecture, as shown in Figure 5. The first layer is trained to predict whether a nucleotide sequence is an enhancer (1) or not (0). The second layer of the proposed model is trained to describe whether an enhancer is a strong (1) or weak (0) one.

### 3.6. Training Procedure of SpEnhancer

This study implements the deep learning model’s training through the packages keras version 2.2.4 and tensorflow-gpu version 1.14.0 in the Python programming language version 3.7.7. The working environment is equipped with the GPU card Nvidia GeForce RTX 2060. In a multi-CPU server environment, we use 8-fold cross-validation to determine three sets of hyperparameters in turn: batch size (pBatchSize), the number of neural units in the LSTM layer (pLSTMSize), and the dropout ratio (pDropoutRatio). Moreover, the results obtained on the leave-one-out method are compared with existing research.

The dataset is retrieved from the database [17].

The model uses the Adam [21] optimizer to guide the model training process, and the optimization goal is the prediction accuracy on the validation dataset.

## 4. Conclusions

This proof-of-principle study demonstrates that the SeqPose features generated by the natural language processing (NLP) technologies achieved similar detection performances of enhancers and their enhancing strengths, compared with the existing best models. The experimental data suggests that the genomic sequences may be regarded as the language of lives, and the functional roles of genomic sequences may be investigated through the NLP technologies.

Our experimental data also demonstrates the importance of removing the unassociated positions from training a DNA sequence-based prediction model. The retaining of some positions in the DNA sequences may even reduce the overall model prediction performances. A previous study showed that many deep learning models may be improved by removing the features without contributions to the models [22]. The time-consuming training process of a deep learning model may be sped up by removing the unassociated features.

It is anticipated that more applications of the NLP technologies will be conducted to investigate genomic functional elements.

## Figures and Tables

**Table 1:** Performance comparison of different k-mers on the test dataset. The performance of individual k-mer setting.

k-mer	Acc	Sn	Sp	MCC	AUC
**1-mer**	0.7811	0.7183	0.8387	0.5319	0.8783
**2-mer**	0.8047	0.7254	0.8774	0.5673	0.8781
**3-mer**	0.7912	0.7042	0.8710	0.5383	0.8693
**4-mer**	0.8013	0.7113	0.8839	0.5554	0.8717
**5-mer**	0.7677	0.7887	0.7484	0.5534	0.8637
**6-mer**	0.7778	0.6338	0.9097	0.4870	0.8652
**7-mer**	0.7306	0.6972	0.7613	0.4498	0.8322

**Table 2:** Performance of different SeqPoseDict on the same test set. The first row gives the values of the five performance metrics—including Sn, Sp, MCC, AUC, and Acc. The first column is different SeqPoseDict.

	Acc	Sn	Sp	MCC	AUC
**Res1**	0.7946	0.6620	0.9161	0.5221	0.8827
**Res2**	0.7912	0.8028	0.7806	0.5951	0.8797
**Res3**	0.8114	0.7394	0.8774	0.8774	0.8920
**Res4**	0.7845	0.8099	0.7613	0.5905	0.8827
**Res5**	0.7845	0.8380	0.7355	0.6100	0.8885

**Table 3:** Performance of different hyperparameters on the test dataset. ‘Upper left corner’: When pBatchSize = 16, traverse the collection of pDropoutRatio and pLSTMSize. ‘Upper right corner’: When pBatchSize = 32, traverse the collection of pDropoutRatio and pLSTMSize. ‘Middle left’: When pBatchSize = 64, traverse the collection of pDropoutRatio and pLSTMSize Collection. ‘Middle right’: When pBatchSize = 128, traverse the collections of pDropoutRatio and pLSTMSize. ‘Lower left corner’: When pBatchSize = 256, traverse the collections of pDropoutRatio and pLSTMSize. ‘Bottom right corner’: When pBatchSize = 512, traverse the collections of pDropoutRatio and pLSTMSize. In each subgraph, the first row gives the values of the five prediction performance metrics—i.e., Sn, Sp, MCC, Acc, and AUC. The first column gives the values of each pair pLSTMSize and pDropoutRatio, separated by a comma.

	Sn	Sp	MCC	Acc	AUC		Sn	Sp	MCC	Acc	AUC
**64,0.1**	0.7180	0.8219	0.5161	0.7692	0.8441	**64,0.1**	0.7057	0.8429	0.5173	0.7729	0.8476
**64,0.2**	0.7489	0.8032	0.5384	0.7759	0.8496	**64,0.2**	0.7468	0.7993	0.5327	0.7729	0.8471
**64,0.3**	0.7135	0.8395	0.5224	0.7773	0.8516	**64,0.3**	0.7301	0.7895	0.5061	0.7571	0.8484
**64,0.4**	0.7309	0.8207	0.5307	0.7756	0.8504	**64,0.4**	0.7145	0.8317	0.5172	0.7712	0.8508
**64,0.5**	0.7408	0.7976	0.5252	0.7685	0.8475	**64,0.5**	0.7001	0.8597	0.5205	0.7800	0.8430
**64,0.6**	0.7393	0.8102	0.5307	0.7743	0.8485	**64,0.6**	0.7242	0.8075	0.5129	0.7652	0.8519
**64,0.7**	0.7319	0.8286	0.5366	0.7810	0.8514	**64,0.7**	0.7263	0.8254	0.5282	0.7756	0.8478
**128,0.1**	0.6746	0.8602	0.4908	0.7662	0.8427	**128,0.1**	0.7295	0.8147	0.5250	0.7729	0.8433
**128,0.2**	0.7256	0.7758	0.4894	0.7507	0.8347	**128,0.2**	0.6743	0.8589	0.4970	0.7642	0.8459
**128,0.3**	0.6919	0.8426	0.4983	0.7665	0.8441	**128,0.3**	0.6894	0.8304	0.4946	0.7642	0.8361
**128,0.4**	0.6854	0.8534	0.5019	0.7709	0.8479	**128,0.4**	0.7620	0.7570	0.5219	0.7571	0.8418
**128,0.5**	0.6947	0.8337	0.4982	0.7638	0.8438	**128,0.5**	0.7322	0.7987	0.5187	0.7655	0.8452
**128,0.6**	0.7289	0.8028	0.5154	0.7655	0.8441	**128,0.6**	0.7539	0.7820	0.5302	0.7682	0.8384
**128,0.7**	0.6866	0.8509	0.4990	0.7672	0.8373	**128,0.7**	0.7255	0.7894	0.5015	0.7551	0.8416
**192,0.1**	0.6969	0.7745	0.4674	0.7385	0.8364	**192,0.1**	0.6825	0.8220	0.4753	0.7520	0.8276
**192,0.2**	0.6868	0.8228	0.4821	0.7537	0.8291	**192,0.2**	0.6480	0.8739	0.4752	0.7618	0.8364
**192,0.3**	0.7047	0.8098	0.4913	0.7574	0.8357	**192,0.3**	0.7250	0.8237	0.5255	0.7756	0.8467
**192,0.4**	0.6406	0.8555	0.4547	0.7470	0.8329	**192,0.4**	0.6430	0.8482	0.4576	0.7439	0.8352
**192,0.5**	0.6286	0.8500	0.4427	0.7358	0.8146	**192,0.5**	0.6854	0.8527	0.5000	0.7692	0.8484
**192,0.6**	0.7567	0.7800	0.5312	0.7695	0.8420	**192,0.6**	0.7468	0.7604	0.5105	0.7564	0.8383
**192,0.7**	0.7089	0.8322	0.5186	0.7746	0.8438	**192,0.7**	0.7373	0.7608	0.4924	0.7456	0.8377
	**Sn**	**Sp**	**MCC**	**Acc**	**AUC**		**Sn**	**Sp**	**MCC**	**Acc**	**AUC**
**64,0.1**	0.7199	0.8250	0.5185	0.7709	0.8497	**64,0.1**	0.7152	0.8372	0.5233	0.7773	0.8527
**64,0.2**	0.7377	0.8393	0.5509	0.7877	0.8567	**64,0.2**	0.7191	0.8276	0.5188	0.7729	0.8507
**64,0.3**	0.7427	0.8106	0.5358	0.7756	0.8521	**64,0.3**	0.6868	0.8618	0.5074	0.7732	0.8530
**64,0.4**	0.6896	0.8444	0.4987	0.7665	0.8464	**64,0.4**	0.6722	0.8288	0.4664	0.7483	0.8316
**64,0.5**	0.7403	0.8361	0.5521	0.7884	0.8506	**64,0.5**	0.7152	0.8233	0.5133	0.7689	0.8428
**64,0.6**	0.6898	0.8411	0.4968	0.7631	0.8507	**64,0.6**	0.6995	0.8154	0.4874	0.7577	0.8401
**64,0.7**	0.7265	0.8049	0.5136	0.7642	0.8486	**64,0.7**	0.7136	0.8191	0.5081	0.7665	0.8497
**128,0.1**	0.6909	0.8390	0.4946	0.7648	0.8340	**128,0.1**	0.6920	0.8464	0.5029	0.7709	0.8466
**128,0.2**	0.7260	0.8166	0.5238	0.7722	0.8479	**128,0.2**	0.6973	0.8428	0.5080	0.7692	0.8469
**128,0.3**	0.6754	0.8299	0.4764	0.7544	0.8393	**128,0.3**	0.6719	0.8415	0.4755	0.7547	0.8447
**128,0.4**	0.6765	0.8409	0.4863	0.7540	0.8443	**128,0.4**	0.6916	0.8168	0.4858	0.7534	0.8444
**128,0.5**	0.7060	0.8300	0.5075	0.7675	0.8470	**128,0.5**	0.6994	0.8343	0.5048	0.7668	0.8392
**128,0.6**	0.6852	0.8611	0.5064	0.7732	0.8459	**128,0.6**	0.7085	0.8366	0.5152	0.7712	0.8498
**128,0.7**	0.6918	0.8507	0.5091	0.7702	0.8430	**128,0.7**	0.6962	0.8189	0.4929	0.7598	0.8518
**192,0.1**	0.6767	0.8462	0.4860	0.7631	0.8378	**192,0.1**	0.6529	0.8787	0.4824	0.7679	0.8488
**192,0.2**	0.6848	0.8393	0.4939	0.7618	0.8480	**192,0.2**	0.6793	0.8445	0.4884	0.7625	0.8345
**192,0.3**	0.6654	0.8538	0.4768	0.7584	0.8283	**192,0.3**	0.7350	0.8156	0.5325	0.7749	0.8528
**192,0.4**	0.6989	0.8349	0.5050	0.7658	0.8437	**192,0.4**	0.6592	0.8623	0.4781	0.7594	0.8444
**192,0.5**	0.6812	0.8499	0.4933	0.7638	0.8472	**192,0.5**	0.6046	0.8926	0.4439	0.7480	0.8441
**192,0.6**	0.6801	0.8374	0.4836	0.7574	0.8313	**192,0.6**	0.7090	0.7949	0.4944	0.7480	0.8405
**192,0.7**	0.7044	0.8305	0.5065	0.7665	0.8422	**192,0.7**	0.7177	0.8210	0.5145	0.7675	0.8467
	**Sn**	**Sp**	**MCC**	**Acc**	**AUC**		**Sn**	**Sp**	**MCC**	**Acc**	**AUC**
**64,0.1**	0.6857	0.8343	0.4910	0.7598	0.8363	**64,0.1**	0.6857	0.7948	0.4581	0.7433	0.8242
**64,0.2**	0.6979	0.8412	0.5052	0.7702	0.8410	**64,0.2**	0.6514	0.8488	0.4591	0.7483	0.8217
**64,0.3**	0.6923	0.8483	0.5027	0.7706	0.8422	**64,0.3**	0.6614	0.8005	0.4319	0.7338	0.8102
**64,0.4**	0.6840	0.8524	0.4975	0.7682	0.8436	**64,0.4**	0.6781	0.7835	0.4398	0.7315	0.8109
**64,0.5**	0.7304	0.8148	0.5252	0.7736	0.8460	**64,0.5**	0.6705	0.8206	0.4593	0.7456	0.8203
**64,0.6**	0.7113	0.8147	0.5040	0.7618	0.8433	**64,0.6**	0.6490	0.7762	0.3986	0.7113	0.8037
**64,0.7**	0.7132	0.8421	0.5245	0.7746	0.8538	**64,0.7**	0.6253	0.8211	0.4091	0.7217	0.8011
**128,0.1**	0.6982	0.8451	0.5123	0.7702	0.8494	**128,0.1**	0.6601	0.8469	0.4701	0.7534	0.8383
**128,0.2**	0.7124	0.8149	0.5069	0.7675	0.8423	**128,0.2**	0.6412	0.8730	0.4655	0.7564	0.8237
**128,0.3**	0.6600	0.8739	0.4869	0.7689	0.8400	**128,0.3**	0.6770	0.8292	0.4736	0.7527	0.8303
**128,0.4**	0.6407	0.8799	0.4708	0.7581	0.8456	**128,0.4**	0.7036	0.8361	0.5097	0.7675	0.8444
**128,0.5**	0.6726	0.8494	0.4857	0.7581	0.8469	**128,0.5**	0.7253	0.8013	0.5114	0.7621	0.8374
**128,0.6**	0.6995	0.8190	0.4900	0.7591	0.8343	**128,0.6**	0.6967	0.8230	0.4887	0.7601	0.8281
**128,0.7**	0.7194	0.8276	0.5228	0.7732	0.8418	**128,0.7**	0.6680	0.8668	0.4912	0.7679	0.8455
**192,0.1**	0.7208	0.7946	0.4964	0.7567	0.8375	**192,0.1**	0.7118	0.8042	0.4961	0.7564	0.8404
**192,0.2**	0.6794	0.8020	0.4556	0.7419	0.8239	**192,0.2**	0.7025	0.7904	0.4719	0.7473	0.8363
**192,0.3**	0.6893	0.7819	0.4526	0.7355	0.8287	**192,0.3**	0.6949	0.8085	0.4779	0.7520	0.8358
**192,0.4**	0.6651	0.8435	0.4715	0.7537	0.8392	**192,0.4**	0.7163	0.7901	0.4908	0.7524	0.8314
**192,0.5**	0.6942	0.8122	0.4836	0.7527	0.8317	**192,0.5**	0.6893	0.8321	0.4885	0.7621	0.8426
**192,0.6**	0.6847	0.8196	0.4739	0.7476	0.8278	**192,0.6**	0.6970	0.8009	0.4750	0.7507	0.8334
**192,0.7**	0.6840	0.8309	0.4816	0.7567	0.8436	**192,0.7**	0.6688	0.8532	0.4808	0.7625	0.8395

**Table 4:** Comparison of the results of the leave-one-out method on the training dataset between spEnhancer and the three existing methods.

	Methods	Acc	Sn	Sp	MCC	AUC
enhancers vs non-enhancers	spEnhancer	0.7793	0.7082	0.8504	0.5227	0.8468
	iEnhancer-EL	0.7803	0.7567	0.8039	0.5613	0.8547
	iEnhancer-2L	0.7689	0.7809	0.7588	0.5400	0.8500
	EnhancerPred	0.7318	0.7257	0.7379	0.4636	0.8082
strong enhancers vs weak enhancers	spEnhancer	0.6413	0.8503	0.3052	0.2105	0.6148
	iEnhancer-EL	0.6503	0.6900	0.6105	0.3149	0.6957
	iEnhancer-2L	0.6193	0.6221	0.6182	0.2400	0.6600
	EnhancerPred	0.6206	0.6267	0.6146	0.2413	0.6601

**Table 5:** Comparison of results between spEnhancer and the three existing methods on the independent test dataset.

	Methods	Acc	Sn	Sp	MCC	AUC
enhancers vs non-enhancers	spEnhancer	0.7725	0.8300	0.7150	0.5793	0.8235
	iEnhancer-ECNN	0.7690	0.7850	0.7520	0.5370	0.8320
	iEnhancer-EL	0.7475	0.7100	0.7850	0.4964	0.8173
	iEnhancer-2L	0.7300	0.7100	0.7500	0.4604	0.8062
	EnhancerPred	0.7400	0.7350	0.7450	0.4800	0.8013
strong enhancers vs weak enhancers	spEnhancer	0.6200	0.9100	0.3300	0.3703	0.6253
	iEnhancer-ECNN	0.6780	0.7910	0.7480	0.3680	0.7480
	iEnhancer-EL	0.6100	0.5400	0.6800	0.2222	0.6801
	iEnhancer-2L	0.6050	0.4700	0.7400	0.2181	0.6678
	EnhancerPred	0.5500	0.4500	0.6500	0.1021	0.5790

**Table 6:** Comparison of the spEnhancer model performances using different dimensions of word vectors on the independent test dataset. The first column gives the binary classification problem. The column “WV” is the dimension of the word vector. The other five columns give the prediction performances Acc, Sn, Sp, MCC, and AUC.

	Word Vector Dimension	Acc	Sn	Sp	MCC	AUC
enhancers vs. non-enhancers	12	0.7085	0.8550	0.5606	0.4943	0.8177
	24	0.6658	0.9150	0.4141	0.4655	0.8094
	48	0.7538	0.8150	0.6919	0.5408	0.8167
	96	0.7060	0.8650	0.5455	0.4971	0.7359
	192	0.7186	0.7650	0.6717	0.4580	0.8078
	394	0.7337	0.8400	0.6263	0.5253	0.8172
	768 (this study)	0.7725	0.8300	0.7150	0.5793	0.8235
	1536	0.7764	0.7550	0.7980	0.5408	0.8281
strong enhancers vs. weak enhancers	12	0.5550	0.4300	0.6800	0.0984	0.6351
	24	0.5000	1.0000	0.0000	0.0000	0.6324
	48	0.6000	0.7100	0.4900	0.2265	0.6342
	96	0.6250	0.8000	0.4500	0.3101	0.6275
	192	0.5700	0.6700	0.4700	0.1565	0.6279
	394	0.5950	0.7100	0.4800	0.2165	0.5987
	768 (this study)	0.6200	0.9100	0.3300	0.3703	0.6253
	1536	0.5800	0.8700	0.2900	0.2469	0.5972

## Data Availability

The Python source code and data can be obtained from the data folder in the link http://www.healthinformaticslab.org/supp/resources.php, accessed on 23 October 2020.
